# HIV Infection Is Associated With Downregulation of BTLA Expression on *Mycobacterium tuberculosis*-Specific CD4 T Cells in Active Tuberculosis Disease

**DOI:** 10.3389/fimmu.2019.01983

**Published:** 2019-08-21

**Authors:** Morgan S. Barham, Deborah A. Abrahams, Jeremiah Khayumbi, Joshua Ongalo, Joan Tonui, Angela Campbell, Marwou de Kock, Samuel Gurrion Ouma, Felix Hayara Odhiambo, Willem A. Hanekom, Neel R. Gandhi, Cheryl L. Day

**Affiliations:** ^1^Emory Vaccine Center, Emory University, Atlanta, GA, United States; ^2^South African Tuberculosis Vaccine Initiative, School of Child and Adolescent Health, Institute of Infectious Diseases and Molecular Medicine, University of Cape Town, Cape Town, South Africa; ^3^Center for Global Health Research, Kenya Medical Research Institute, Kisumu, Kenya; ^4^Department of Epidemiology, Rollins School of Public Health, Emory University, Atlanta, GA, United States; ^5^Division of Infectious Diseases, Department of Medicine, Emory University School of Medicine, Atlanta, GA, United States; ^6^Department of Microbiology and Immunology, Emory University School of Medicine, Atlanta, GA, United States

**Keywords:** HIV, *Mycobacterium tuberculosis*, LTBI, active TB disease, CD4 T cell, BTLA, CTLA-4, PD-1

## Abstract

Nearly a quarter of the global population is infected with *Mycobacterium tuberculosis* (Mtb), with 10 million people developing active tuberculosis (TB) annually. Co-infection with human immunodeficiency virus (HIV) has long been recognized as a significant risk factor for progression to TB disease, yet the mechanisms whereby HIV impairs T cell-mediated control of Mtb infection remain poorly defined. We hypothesized that HIV infection may promote upregulation of inhibitory receptors on Mtb-specific CD4 T cells, a mechanism that has been associated with antigen-specific T cell dysfunction in chronic infections. Using cohorts of HIV-infected and HIV-uninfected individuals with latent Mtb infection (LTBI) and with active TB disease, we stimulated peripheral blood mononuclear cells (PBMC) for 6 hours with Mtb peptide pools and evaluated co-expression profiles of the inhibitory receptors BTLA, CTLA-4, and PD-1 on IFN-γ^+^/TNF-α^+^ Mtb-specific CD4 T cells. Mtb-specific CD4 T cells in all participant groups expressed predominately either one or no inhibitory receptors, unlike cytomegalovirus- and HIV-specific CD4 T cells circulating in the same individuals, which were predominately CTLA-4^+^PD-1^+^. There were no significant differences in inhibitory receptor expression profiles of Mtb-specific CD4 T cells between HIV-uninfected and HIV-infected individuals with LTBI. Surprisingly, BTLA expression, both alone and in combination with CTLA-4 and PD-1, was markedly downregulated on Mtb-specific CD4 T cells in HIV-infected individuals with active TB. Together, these data provide novel evidence that the majority of Mtb-specific CD4 T cells do not co-express multiple inhibitory receptors, regardless of HIV infection status; moreover, they highlight a previously unrecognized role of BTLA expression on Mtb-specific CD4 T cells that could be further explored as a potential biomarker of Mtb infection status, particularly in people living with HIV, the population at greatest risk for development of active TB disease.

## Introduction

*Mycobacterium tuberculosis* (Mtb) is the infectious agent that causes tuberculosis (TB) disease (1). TB is the leading cause of death due to a single infectious agent and has remained one of the top 10 causes of death worldwide for decades (1). In 2017, 10 million new cases of TB disease were reported, resulting in 1.6 million deaths (1). An estimated 1.7 billion people, representing nearly a quarter of the world's population, are latently infected with Mtb and therefore at risk for developing active TB disease (2). Although the precise immune correlates of protection against TB have not been defined, co-infection with human immunodeficiency virus (HIV) is the single greatest risk factor for reactivation from latent Mtb infection (LTBI) to active TB disease (1, 3). Worldwide, ~9% of new reported TB cases occur in people living with HIV, of which 72% live in Africa (1).

Infection with HIV induces immune suppression and depletion of CD4 T cells, which play a critical role in limiting Mtb bacterial growth and reducing progression to active TB disease (4). Mtb-specific CD4 T cells in HIV-infected individuals exhibit elements of immune dysfunction, including impaired proliferative capacity, heightened immune activation and cell death (5), and intermediate differentiated effector memory profiles (6). IL-2 producing Mtb-specific CD4 T cells have been inversely correlated with HIV viral load in individuals with LTBI (6), and decreased frequencies of cytokine-producing Mtb-specific CD4 T cell subsets in HIV-infected individuals (5, 7–10). Other studies have demonstrated that Mtb-specific CD4 T cells are depleted early after HIV seroconversion (11) and that Mtb-specific CD4 T cells may be preferentially infected by HIV (12). Although HIV co-infection clearly disrupts protective immunity to Mtb, the precise mechanisms whereby HIV impairs Mtb-specific T cell immunity and accelerates progression to TB disease have not been fully elucidated.

Ag-specific T cell dysfunction is a well-described feature of chronic infections, including HIV, with upregulation of negative regulatory receptors on Ag-specific T cells described as one mechanism contributing to inhibition of T cell activation and effector functions such as cytokine production, cytotoxicity, and proliferation (13). In mice with chronic lymphocytic choriomeningitis virus (LCMV) infection, transcriptional profiling of dysfunctional or “exhausted” LCMV-specific CD8 T cells identified inhibitory receptors with sustained expression at high levels on dysfunctional T cells, including PD-1, CTLA-4, 2B4, CD160, and LAG-3 (14, 15). While T cell dysfunction in chronic infections was initially described in Ag-specific CD8 T cells, Ag-specific CD4 T cells also exhibit functional impairment and high expression of inhibitory receptors in the setting of persistent Ag stimulation (16). Similar to CD8 T cells, Ag-specific CD4 T cell in chronic infections express high levels of PD-1 and CTLA-4, as well as B and T lymphocyte attenuator (BTLA), which is upregulated on Ag-specific CD4 T cells but not Ag-specific CD8 T cells in chronic infection (17, 18). Moreover, co-expression of multiple inhibitory receptors is associated with greater severity of Ag-specific T cell dysfunction (17), thus providing evidence of the additive and detrimental effect of co-expression of multiple inhibitory receptors on Ag-specific T cell function.

T cell dysfunction associated with increased expression of inhibitory receptors has been well-described in chronic HIV infection (19), and expression of inhibitory receptors, including PD-1 and CTLA-4, on HIV-specific T cells correlates with viral load and absolute CD4 T cell count (20, 21), important parameters of HIV disease progression. Moreover, HIV-specific CD4 T cells co-express combinations of PD-1, CTLA-4 and LAG-3 (21–23), which correlates with viral load and decreases after suppression of HIV viremia by antiretroviral therapy (22). Blockade of PD-1 and CTLA-4 signaling in HIV-specific CD4 T cells enhances cytokine production and proliferation (20, 21, 23, 24), thus providing further evidence of the relationship between inhibitory receptor expression and HIV-specific CD4 T cell dysfunction. Although systemic immune activation and upregulation of inhibitory receptors on HIV-specific CD4 T cells are well-described in HIV-infected individuals, it is currently unclear whether HIV infection is also associated with increased expression of inhibitory receptors on CD4 T cells specific for other co-infections, such as Mtb, which may subsequently impair T cell-mediated control of infection.

Given that HIV infection is associated with upregulation of inhibitory receptors on CD4 T cells, and that the risk of developing active TB disease is more than 20-fold higher in HIV-infected individuals, compared with HIV-uninfected individuals (1, 3), we sought to determine if inhibitory receptors are upregulated on Mtb-specific CD4 T cells in individuals co-infected with Mtb and HIV, which may ultimately contribute to impairment of Mtb-specific CD4 T cell functional capacity and progression to active TB disease. Using cohorts of HIV-infected and HIV-uninfected individuals with LTBI and active TB in South Africa and Kenya, two high TB burden countries (1), we conducted a thorough examination of inhibitory receptor expression on Ag-specific CD4 T cells. We evaluated expression patterns of BTLA, CTLA-4, and PD-1, three inhibitory receptors on T cells that are members of the immunoglobuin (Ig) superfamily and have reported to be upregulated on Ag-specific CD4 T cells in chronic infections (18, 21–23). Furthermore, we compared inhibitory receptor expression profiles of Mtb-specific CD4 T cells to that of human cytomegalovirus (HCMV)- and HIV-specific CD4 T cells within the same individual.

## Materials and Methods

### Study Participants and Sample Collection

Blood samples were collected from individuals ≥18 years of age living in Western Kenya and in the Western Cape province of South Africa. Study participants included HIV-uninfected and HIV-infected adults with LTBI or active TB disease (5, 25). Individuals with LTBI included in the study had a positive QuantiFERON-TB Gold (QFT) test result and no previous history of active TB; LTBI participants were asymptomatic for TB, with no cough, weight loss, night sweats, or fever. Participants with active TB disease were acid-fast bacilli (AFB) sputum smear-positive or Xpert *MTB*/RIF assay-positive, with pulmonary TB disease confirmed by a positive mycobacterial sputum culture. Blood was collected from patients with TB disease within the first 7 days of starting the standard 6-month course of TB treatment. Serologic testing for HIV antibodies was done for all individuals using the Alere Determine HIV-1/2 Ag/Ab Combo test. Plasma HIV viral load and CD4 T cell counts were measured for HIV-infected individuals; viral load results were not available from three HIV-infected participants with active TB. All HIV-infected individuals with LTBI had >200 CD4 T cells/μl. With the exception of 4 individuals with active TB disease, all HIV-infected participants were antiretroviral therapy-naïve at the time of analysis. Blood samples from all participants were collected in sodium heparin tubes for isolation of peripheral blood mononuclear cells (PBMC) for analysis of Ag-specific CD4 T cells, as described below.

### Ethics Statement

This study was conducted in accordance with the principles expressed in the Declaration of Helsinki. All subjects provided written informed consent for participation in the study, which was approved by the Human Research Ethics Committee at the University of Cape Town, the Western Cape Province Department of Health, the Kenya Medical Research Institute Scientific and Ethics Review Unit, and the Emory University Institutional Review Board.

### PBMC Isolation and Antigen Stimulation

Within 4 h of blood collection, PBMC were isolated from heparinized whole blood via density gradient centrifugation using Ficoll-Hypaque (Sigma-Aldrich) and then cryopreserved and stored in LN_2_ until use. Cryopreserved PBMC were thawed in a 37°C water bath and immediately added to RPMI 1640 (Cellgro) containing deoxyribonuclease I (DNase, 10 μg/ml, Sigma-Aldrich). Cells were centrifuged at 2,000 RPM for 5 min at 25°C, resuspended in RPMI, and centrifuged again under the same conditions. Cells were suspended in R10 media (RPMI 1640 supplemented with 10% heat-inactivated fetal calf serum [FCS], 100 U/ml penicillin, 100 μg/ml streptomycin, and 2 mM L-glutamine) and rested for a minimum of 4 h 37°C and 5% CO_2_. After resting, cells were stimulated with the following antigens: pooled, overlapping 15-mer peptides corresponding to the sequences of CFP-10 (22 peptides, 1 μg/ml/peptide), ESAT-6 (21 peptides, 1 μg/ml/peptide), and human CMV (HCMV) pp65 (138 peptides, 1 μg/ml/peptide). Pooled, overlapping 15-mer peptides comprising HIV-1 consensus A Gag (122 peptides, 1 μg/ml/peptide) and HIV-1 consensus C Gag (121 peptides, 1 μg/ml/peptide) were used to stimulate PBMC from Kenyan and South African HIV-infected individuals, respectively. The CFP-10 and ESAT-6 peptide pools were obtained through BEI Resources, NIAID, NIH (catalog numbers NR-50712 and NR-50711, respectively). The HCMV pp65 peptide pool was obtained from the National Institutes of Health AIDS Reagent Program, Division of AIDS, NIAID, NIH (26–28). The HIV Gag peptide pools were obtained through the NIH AIDS Reagent Program, Division of AIDS, NIAID, NIH: HIV-1 Subtype C (DU422) Gag Peptide Set and HIV-1 Consensus A Gag Peptides—Complete Set. PBMC were stimulated with staphylococcal enterotoxin B (SEB; 1 μg/ml; Sigma-Aldrich) as a positive control. PBMC incubated with no Ag were used as a negative control. Brefeldin A (10 μg/ml; Sigma-Aldrich) was added after a 1-h incubation at 37°C and the incubation continued for an additional 5 h at 37°C.

### Antibodies and Intracellular Cytokine Staining (ICS) Assay

Stimulated cells were washed with PBS and stained for 20 min at room temperature with Zombie NIR? Fixable Viability Dye (BioLegend). Cells were surface stained for 30 min at room temperature with anti-CD3 Brilliant Violet 711 (UCHT1; BD Horizon), anti-CD4 Brilliant Violet 570 (RPA-T4; BioLegend), anti-CD8 PerCP-Cy5.5 (SK1; BioLegend), anti-PD-1 PE (EH12.2H7; BioLegend), and anti-BTLA APC (MIH26; BioLegend). Cells were washed with PBS containing 1% FCS and fixed with FACS Lysing Solution (BD Biosciences), then washed with Perm/Wash Buffer (BD Biosciences). Cells were stained intracellularly for 30 min at room temperature with anti-IFN-γ FITC (B27; BD Pharmingen), anti-TNF-α Brilliant Violet 421 (Mab11; BioLegend), and anti-CD152 PE-CF594 (BNI3; BD Horizon), washed with Perm/Wash Buffer (BD Biosciences) and suspended in PBS prior to acquisition.

### Flow Cytometry and Data Analysis

Cells were acquired on a BD LSRII flow cytometer with BD FACSDiva software (v8.0) and analyzed using FlowJo software (v9.7.6; Tree Star). Compensation was calculated using single-stained anti-mouse Ig, κ beads (BD Biosciences). Doublet cell populations were excluded by plotting forward scatter area vs. forward scatter height. Viable lymphocytes were defined as Zombie NearIR-low cells. Combinations of cytokine-producing cells were determined using Boolean gating in FlowJo. The flow cytometry gating strategy is indicated in Figure S1.

### Data Analysis and Statistics

Only individuals with a positive response to a given Ag were included in the phenotypic analyses of Ag-specific CD4 T cells. CD4 T cells producing TNF-α and/or IFN-γ were used to determine Ag-specific responses via the Bayesian mathematical model, MIMOSA (Mixture Models for Single Cell Assays) (29) to account for cell counts and background cytokine secretion. TNF-α- and IFN-γ-producing CD4 T cells with a positive response rate >75% and a false discover rate (fdr/*q*-value) < 3% were considered positive responses. R programming software was used to perform statistical analyses of frequencies and phenotype of Ag-specific CD4 T cells (30). Differences between two groups were evaluated using a non-parametric Mann-Whitney test. Differences between three or more groups were first evaluated using a non-parametric Kruskal-Wallis test, with *p*-values adjusted for multiple comparisons using Dunn's *post-test*. *P*-values of < 0.05 was considered to be statistically significant.

## Results

### Study Participants

Blood samples were collected from 32 HIV-uninfected and 22 HIV-infected participants with LTBI, and from 37 HIV-uninfected and 19 HIV-infected participants with pulmonary TB disease enrolled in Western Kenya and in the Western Cape Province of South Africa (Table 1). The median CD4 T cell count of HIV-infected participants with LTBI was 562 cells/μl; the median viral load was 11,710 HIV RNA copies/ml plasma. The median CD4 T cell count of HIV-infected participants with active TB disease was 420 cells/μl; the median viral load was 6,350 HIV RNA copies/ml plasma.

**Table 1:** Characteristics of study participants.

**Participant group**	***n***	**Age, y^a^ (IQR)**	**Sex (% male)**	**CD4 count, cells/μl^b^ (IQR)**	**HIV viral load, copies RNA/ml plasma^b^ (IQR)**
LTBI/HIV–	32	32 (20–41)	38	N/A	N/A
LTBI/HIV+	22	35 (28–44)	18	562 (432–595)	11,710 (2,855–30,586)
TB/HIV–	37	31 (22–37)	68	N/A	N/A
TB/HIV+	19	36 (29–42)^c^	42	420 (261–614)	6,350 (256–36,368)*^d^*

a *Value denotes median age in years*.

b *Values denote median*.

c *p < 0.05, compared with TB/HIV–*.

d *Viral load not available for 3 TB/HIV+ participants*.

*IQR, interquartile range; N/A, not applicable*.

### Frequencies of Mtb-Specific CD4 T Cells Producing IFN-γ and/or TNF-α Are Similar in HIV-Infected and HIV-Uninfected Individuals

**Figure 1:**
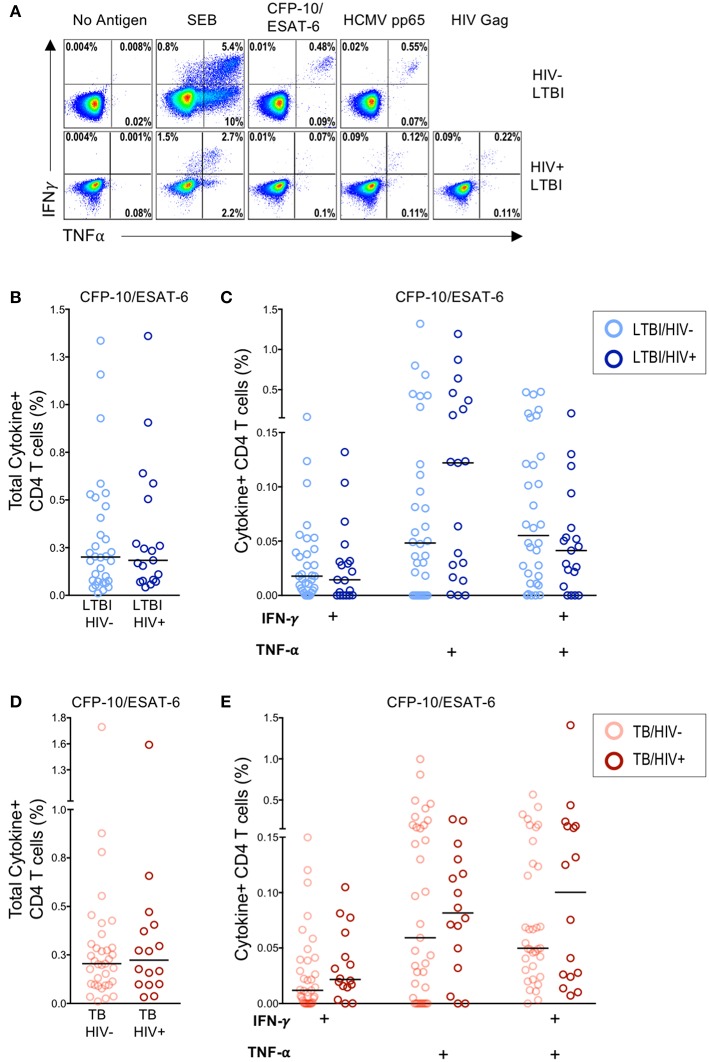
Similar frequencies of Mtb-specific T cells producing IFN-γ and TNF-α in HIV-infected and HIV-uninfected individuals with latent and active TB. PBMC from HIV-uninfected (*n* = 32) and HIV-infected (*n* = 22) individuals with LTBI and HIV-uninfected (*n* = 37) and HIV-infected (*n* = 19) individuals with active TB were incubated for 6 h in media alone (negative control) or stimulated with Ags (CFP-10/ESAT-6 peptide pools, HCMV pp65 peptide pool, HIV Gag peptide pool, and SEB). Intracellular expression of IFN-γ and TNF-α was measured by flow cytometry. **(A)** Representative flow cytometry data from an HIV-uninfected individual with LTBI (top row), and an HIV-infected individual with LTBI (bottom row). Plots are shown gated on live CD3^+^CD4^+^ lymphocytes. **(B)** Total frequency of cytokine^+^ Mtb-specific CD4 T cells from HIV-uninfected and HIV-infected individuals with LTBI. **(C)** Frequencies of the indicated cytokine^+^ subsets of Mtb-specific CD4 T cells from HIV-uninfected and HIV-infected individuals with LTBI. **(D)** Total frequency of cytokine^+^ Mtb-specific CD4 T cells from HIV-uninfected and HIV-infected individuals with active TB. **(E)** Frequencies of the indicated cytokine^+^ subsets of Mtb-specific CD4 T cells in HIV-uninfected and HIV-infected individuals with active TB. Horizontal lines represent the median. Data are shown after subtraction of background cytokine production in the unstimulated negative control condition. Differences in the frequencies of each cytokine^+^ T cell population between HIV-uninfected and HIV-infected individuals were assessed using a Mann Whitney *U* test.

Infection with HIV is associated with CD4 T cell depletion and dysfunction. To further characterize the effect of HIV infection on the frequency of Mtb-specific CD4 T cells, we used a flow cytometry-based ICS assay to measure Ag-specific CD4 T cell responses in HIV-infected and HIV-uninfected individuals with LTBI and with active TB disease. PBMC were stimulated for 6 h with the immunodominant Mtb Ags CFP-10 and ESAT-6 and viral Ags (HCMV pp65 and HIV Gag peptide pools); Ag-specific CD4 T cells were identified by production of IFN-γ and TNF-α (Figure 1A). No significant differences in the frequency of CFP-10/ESAT-6-specific CD4 T cells producing IFN-γ and/or TNF-α were observed between HIV-uninfected and HIV-infected individuals with LTBI (Figures 1B,C) or active TB disease (Figures 1D,E). The frequencies of CD4 T cells producing IFN-γ and/or TNF-α following stimulation with HCMV pp65 and SEB were also similar between HIV-infected and HIV-uninfected individuals with latent and active TB (Figure S2). These data indicate that circulating CFP-10/ESAT-6-specific CD4 T cells with the capacity to produce the Th1 effector cytokines IFN-γ and TNF-α are not significantly depleted in HIV-infected individuals with either LTBI or active TB, compared with HIV-uninfected individuals.

### Active TB Disease Is Associated With Reduced Inhibitory Receptor Co-expression on Total CD4 T Cells

**Figure 2:**
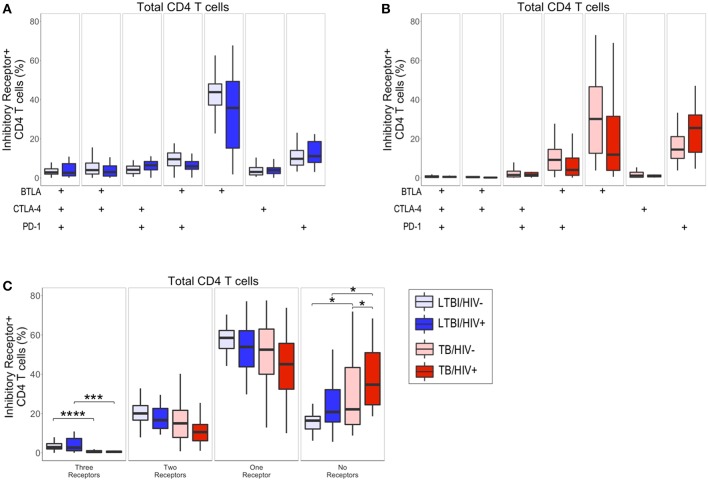
Active TB disease is associated with reduced inhibitory receptor co-expression on total CD4 T cells. PBMC from HIV-uninfected and HIV-infected individuals with LTBI (*n* = 32 and *n* = 22, respectively) and active TB (*n* = 37 and *n* = 19, respectively) were analyzed by flow cytometry for expression of the inhibitory receptors BTLA, CTLA-4, and PD-1 on total CD4 T cells. **(A,B)** The frequencies of total CD4 T cells expressing each combination of BTLA, CTLA-4, and PD-1 are shown for individuals with LTBI **(A)** and active TB **(B)**. **(C)** Summary data representing the proportion of total CD4 T cells expressing three, two, one, or no inhibitory receptors from HIV-uninfected and HIV-infected individuals with LTBI and active TB. Boxes represent the median and interquartile ranges; whiskers represent the 5th and 95th percentiles. Differences in the proportion of CD4 T cells expressing each inhibitory receptor population between HIV-uninfected and HIV-infected individuals **(A,B)** were assessed using a Mann-Whitney *U* test. Differences among groups in the number of inhibitory receptors expressed by CD4 T cells in panel C were assessed using a Kruskal-Wallis test, with *p*-values adjusted for multiple comparisons using Dunn's *post-test*. ^*^p < 0.05; ^***^p < 0.001; ^****^p < 0.0001.

In addition to the frequency of cytokine-producing T cells, expression profiles of multiple types of receptors are indicative of functional status of Ag-specific T cells. Given that infection with HIV has been shown to lead to modulation of T cell phenotypic profiles (31), we next determined the effect of HIV infection on the total CD4 T cell expression profiles of BTLA, CTLA-4, and PD-1 in individuals with LTBI and with active TB, using a Boolean gating strategy (Figure 2). In individuals with LTBI, the predominant population consisted of CD4 T cells expressing BTLA alone, regardless of HIV infection status (Figure 2A). In individuals with active TB, CD4 T cells predominately express either BTLA alone or PD-1 alone (Figure 2B).

Although there were no significant differences in the frequency of CD4 T cell subsets expressing each combination of BTLA, CTLA-4, and PD-1 between HIV-infected and HIV-uninfected individuals (Figures 2A,B), we did observe significant differences in the overall total number of inhibitory receptors expressed by CD4 T cells in individuals with LTBI and active TB (Figure 2C). Individuals with active TB had significantly lower frequencies of CD4 T cells expressing three inhibitory receptors, compared with individuals with LTBI. The decrease in CD4 T cells expressing three inhibitory receptors in individuals with active TB corresponded to a significant increase in their proportion of CD4 T cells lacking co-expression of the three inhibitory receptors measured (Figure 2C). Importantly, the expansion of CD4 T cells expressing no inhibitory receptors in active TB, compared with LTBI, was evident in both HIV-infected and HIV-uninfected individuals. Taken together, these data suggest that TB disease status impacts CD4 T cell inhibitory receptor expression profiles and is associated with expansion of circulating CD4 T cells that lack co-expression of BTLA, CTLA-4, and PD-1.

### Mtb-Specific CD4 T Cell Inhibitory Receptor Expression Profiles Are Modulated in HIV-Infected Individuals With Active TB

**Figure 3:**
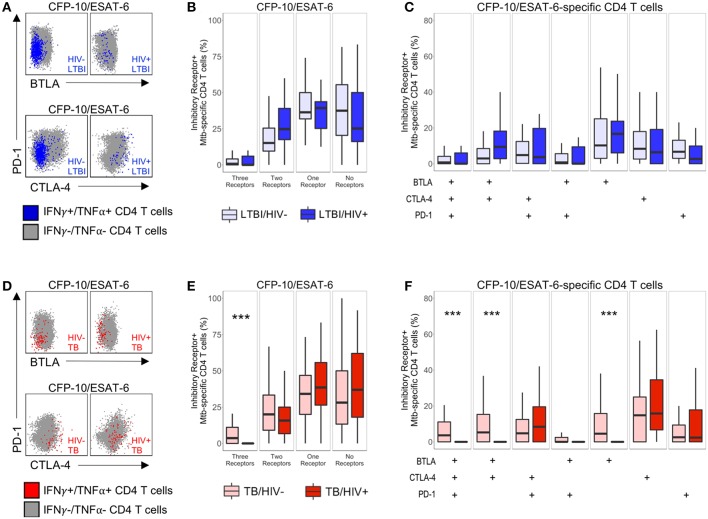
Modulation of Mtb-specific CD4 T cell inhibitory receptor expression profiles in HIV-infected individuals with active TB. PBMC from HIV-uninfected and HIV-infected individuals were stimulated with CFP-10 and ESAT-6 peptide pools and evaluated for expression of IFN-γ and TNF-α by flow cytometry, as described in Figure 1. Mtb-specific CD4 T cells meeting the criteria for a positive response (see Materials and Methods) were evaluated for expression of BTLA, CTLA-4, and PD-1. **(A,D)** Representative flow cytometry data of BTLA, CTLA-4, and PD-1 expression are shown from an HIV-uninfected and HIV-infected individual with LTBI **(A)** and active TB **(D)**. Flow plots are shown gated on live CD3^+^CD4^+^ lymphocytes. Gray dots represent the total cytokine-negative CD4 T cell population; blue and red dots represent IFN-γ and/or TNF-α producing CD4 T cells from individuals with LTBI and TB, respectively. **(B,E)** Composite data from individuals with LTBI **(B)** and TB **(E)** indicating the proportion of CFP-10/ESAT-6-specific CD4 T cells expressing three, two, one or no inhibitory receptors. **(C,F)** Composite data from individuals with LTBI **(C)** and TB **(F)** indicating the proportion of CFP-10/ESAT-6-specific CD4 T cells expressing the indicated combinations of BTLA, PD-1, and CTLA-4 (LTBI/HIV^−^, *n* = 27; LTBI/HIV^+^, *n* = 17; TB/HIV^−^, *n* = 35; TB/HIV^+^, *n* = 16). Boxes represent the median and interquartile ranges; whiskers represent the 5th and 95th percentiles. Differences in the proportions of Mtb-specific CD4 T cells expressing the indicated inhibitory receptors between HIV-uninfected and HIV-infected individuals were assessed using a Mann-Whitney *U* test. ^***^p < 0.001.

Next, we sought to determine whether co-infection with HIV significantly modifies inhibitory receptor expression profiles of Mtb-specific CD4 T cells. To do so, we used our ICS assay to analyze expression of BTLA, CTLA-4, and PD-1 on CD4 T cells producing IFN-γ and/or TNF-α following stimulation with CFP-10 and ESAT-6 peptide pools (Figures 3A,D). MIMOSA was used to define cytokine-positive Mtb-specific CD4 T cell responses for further phenotypic analysis of BTLA, CTLA-4, and PD-1 expression (29).

We first determined the total number of inhibitory receptors expressed by Mtb-specific CD4 T cells in individuals with LTBI. In both groups of individuals with LTBI, the majority of Mtb-specific CD4 T cells expressed either no inhibitory receptors or only one inhibitory receptor (Figure 3B). There were no significant differences in the proportion of Mtb-specific CD4 T cells expressing three, two, one, or no inhibitory receptors in HIV-infected individuals, compared with HIV-uninfected individuals with LTBI (Figure 3B). Similar to individuals with LTBI, the majority of Mtb-specific CD4 T cells in individuals with active TB disease expressed either no or only one inhibitory receptor (Figure 3E). However, HIV-infected individuals with active TB disease had a significantly lower proportion of Mtb-specific CD4 T cells co-expressing all three inhibitory receptors, compared with HIV-uninfected patients with active TB.

We next evaluated the proportion of Mtb-specific CD4 T cells expressing each combination of BTLA, CTLA-4, and PD-1. Among Mtb-specific CD4 T cells that expressed between one and three inhibitory receptors, there was no single dominant subset represented in either HIV-infected or HIV-uninfected individuals with LTBI; moreover, there was no significant difference in the proportion of Mtb-specific CD4 T cells expressing each combination of inhibitory receptors between HIV-infected and HIV-uninfected individuals with LTBI (Figure 3C). By contrast, HIV infection of individuals with active TB disease was associated with a significant decrease in three subsets of Mtb-specific CD4 T cells, compared with HIV-uninfected individuals: BTLA^+^CTLA-4^+^PD-1^+^, BTLA^+^CTLA-4^+^PD-1^−^, and BTLA^+^CTLA-4^−^PD-1^−^ cells (Figure 3F). Together, these data indicate that Mtb-specific CD4 T cell inhibitory receptor expression profiles are significantly modified in the setting of HIV co-infection with active TB, but not LTBI.

### BTLA Expression Is Downregulated on Mtb-Specific CD4 T Cells HIV-Infected Individuals With Active TB

**Figure 4:**
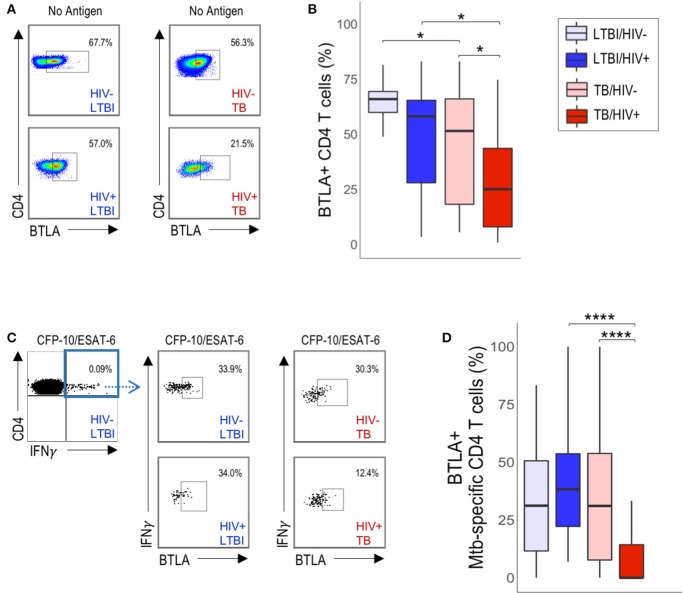
HIV infection is associated with downregulation of BTLA expression on total CD4 T cells and Mtb-specific CD4 T cells in active TB. PBMC from HIV-uninfected and HIV-infected individuals with LTBI and TB were evaluated for BTLA expression by flow cytometry. **(A)** Flow plots are shown gated on live CD3^+^CD4^+^ lymphocytes. **(B)** Summary of BTLA expression on total unstimulated CD4 T cells from HIV-uninfected and HIV-infected individuals with LTBI and active TB. **(C)** Representative flow data of PBMC following stimulation with CFP-10/ESAT-6 peptide pools, as described in Figure 1. Cells were gated on live CD3^+^CD4^+^IFN-γ^+^ T cells, then evaluated for BTLA expression. **(D)** Summary of BTLA expression on CFP-10/ESAT-6-specific CD4 T cells from HIV-uninfected and HIV-infected individuals with LTBI and active TB. Boxes in panels B and D represent the median and interquartile ranges; whiskers represent the 5th and 95th percentiles. Differences in BTLA expression among groups in **(B,D)** were assessed using a Kruskal-Wallis test, with *p*-values adjusted for multiple comparisons using Dunn's *post-test*. ^*^p < 0.05, ^****^p < 0.0001.

A common feature of the three phenotypic subsets of Mtb-specific CD4 T cells that are significantly decreased in HIV-infected individuals with active TB is expression of BTLA, which has previously been reported to be downregulated on T cells in HIV-infected individuals, compared with HIV-uninfected individuals (32). Therefore, we next evaluated whether CD4 T cell expression of BTLA expression alone, independent of PD-1 and CTLA-4 expression, was different between HIV-infected and HIV-uninfected individuals (Figure 4A). We found that BTLA expression was downregulated on total CD4 T cells from HIV-infected individuals with active TB, compared with HIV-uninfected individuals with active TB and with HIV-infected individuals with LTBI (Figure 4B). Although BTLA expression on Mtb-specific CD4 T cells was similar in HIV-infected and HIV-uninfected individuals with LTBI (Figures 4C,D), it was significantly downregulated on Mtb-specific CD4 T cells in HIV-infected individuals with active TB, compared with HIV-uninfected individuals with active TB (Figure 4D). Furthermore, BTLA expression on Mtb-specific CD4 T cells in HIV-infected individuals with active TB was significantly lower than Mtb-specific CD4 T cells in HIV-infected individuals with LTBI (Figure 4D). By contrast, evaluation of CTLA-4 alone and PD-1 alone did not indicate any significant differences in expression levels on either total or Mtb-specific CD4 T cells in HIV-infected and uninfected individuals with either LTBI or active TB (Figure S3). Together, these data provide further evidence that HIV infection is associated with downregulation of BTLA expression on CD4 T cells; moreover, they identify BTLA as a phenotypic marker on Mtb-specific CD4 T cells that is specifically downregulated in HIV-infected individuals with active TB disease, but not LTBI.

To determine whether CD4 T cell expression of BTLA is associated with parameters of HIV disease progression, we analyzed the relationship between CD4 T cell BTLA expression and HIV viral load (Figure S4) and absolute CD4 T cell count in individuals with LTBI and with active TB (Figure S5). We found no significant correlation between HIV viral load and the proportion of either total CD4 or Mtb-specific CD4 T cells expressing BTLA (Figure S4). Additionally, we found no significant correlation between absolute CD4 T cell count and BTLA expression on either total CD4 or Mtb-specific CD4 T cells (Figure S5). These data indicate that CD4 T cell downregulation of BTLA expression in HIV-infected individuals is not directly related to HIV disease progression. Moreover, they suggest that marked downregulation of BTLA expression on Mtb-specific CD4 T cells occurs particularly in the setting of concurrent active TB disease and HIV infection.

### Inhibitory Receptor Expression on Mtb-Specific CD4 T Cells Differs From HCMV- and HIV-Specific CD4 T Cells

**Figure 5:**
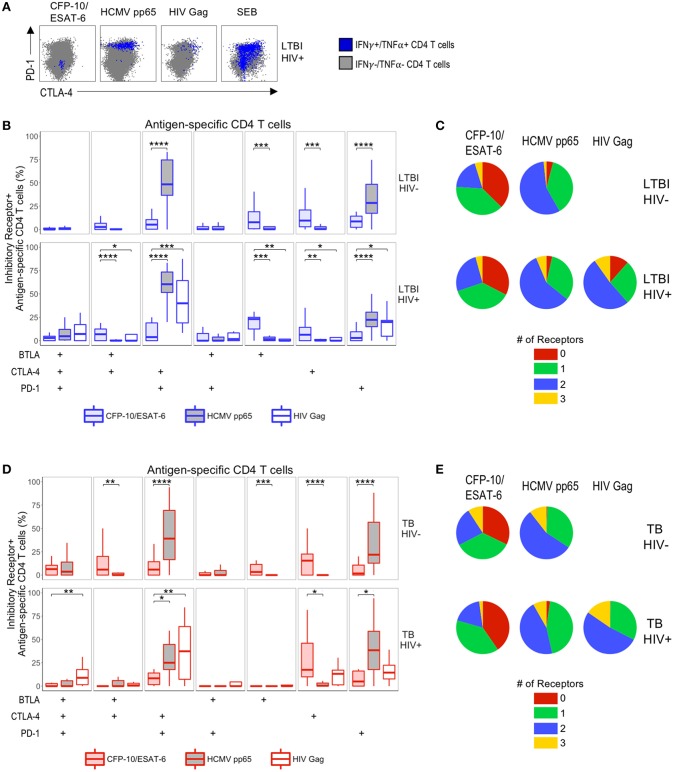
Differential inhibitory receptor expression profiles on Mtb-specific CD4 T cells, compared with HCMV- and HIV-specific CD4 T cells within the same individuals. PBMC were stimulated with CFP-10 and ESAT-6 peptide pools as described in Figure 1, as well as HCMV pp65 peptide pool and HIV Gag peptide pool. Ag-specific CD4 T cells meeting the criteria for a positive response (see Materials and Methods) were evaluated for expression of BTLA, CTLA-4, and PD-1. **(A)** Representative intracellular cytokine staining flow cytometry data from an HIV-infected individual with LTBI. Plots are shown gated on live CD3^+^CD4^+^ lymphocytes. Gray dots represent cytokine-negative CD4 T cells; blue dots represent IFN-γ^+^ and/or TNF-α^+^ CD4 T cells. **(B,C)** Summary data of the proportion of Ag-specific CD4 T cells expressing the indicated subsets of inhibitory receptors **(B)** and the proportion of Ag-specific CD4 T cells expressing three, two, one, or no inhibitory receptors **(C)** in individuals with LTBI (top row, HIV-uninfected LTBI; bottom row, HIV-infected LTBI). **(D,E)** Summary data of the proportion of Ag-specific CD4 T cells expressing the indicated subsets of inhibitory receptors **(D)** and the proportion of Ag-specific CD4 T cells expressing three, two, one, or no inhibitory receptors (E) in individuals with active TB (top row, HIV-uninfected TB; bottom row, HIV-infected TB). Boxes represent the median and interquartile ranges; whiskers represent the 5th and 95th percentiles. Differences between Mtb- and HCMV-specific CD4 T cells in HIV-uninfected individuals **(B,D)** were assessed using a Mann-Whitney *U* test. Differences between Mtb-specific and HCMV- and HIV-specific CD4 T cells in HIV-infected individuals **(B,D)** were assessed using a Kruskal-Wallis test, with *p*-values adjusted for multiple comparisons using Dunn's *post-test*. ^*^p < 0.05; ^**^p < 0.01; ^***^p < 0.001; ^****^p <0.0001.

Previous studies have indicated that expression of PD-1 and CTLA-4 is upregulated on HIV-specific CD4 T cells (21), yet we did not find evidence that HIV co-infection is associated with upregulation of PD-1 and CTLA-4 expression on Mtb-specific CD4 T cells, compared with HIV-uninfected individuals. To further define inhibitory receptor profiles of Mtb-specific CD4 T cells in the setting of HIV infection, we directly compared expression of BTLA, CTLA-4, and PD-1 on circulating Mtb-, HCMV-, and HIV-specific CD4 T cells within the same individual (Figure 5A). HCMV- and HIV-specific CD4 T cells predominately co-expressed CTLA-4 and PD-1 (Figure 5B). By contrast, Mtb-specific CD4 T cells from the same individuals consistently exhibited markedly lower proportions of CTLA-4^+^PD-1^+^ cells and PD-1 single-positive cells, compared with virus-specific CD4 T cells in both HIV-infected and HIV-uninfected individuals with LTBI and active TB (Figures 5B,D). A significantly higher proportion of Mtb-specific CD4 T cells from all four participant groups were CTLA-4 single-positive, compared with HCMV-specific CD4 T cells in the same individuals (Figures 5B,D). With the exception of HIV-infected individuals with active TB disease, in who BTLA is downregulated (Figure 4D), a significantly higher proportion of Mtb-specific CD4 T cells were also BTLA single-positive, compared with HCMV- and HIV-specific CD4 T cells (Figures 5B,D).

Since expression of multiple inhibitory receptors on HIV-specific CD4 T cells has been associated with HIV disease progression (22), we also evaluated the proportion of Mtb-, HCMV- and HIV-specific CD4 T cells expressing three, two, one, or no inhibitory receptors in individuals with LTBI and active TB (Figures 5C,E). In all 4 participant groups, the majority of Mtb-specific CD4 T cells expressed either no or one inhibitory receptor, whereas the majority of HCMV- and HIV-specific CD4 T cells co-expressed two inhibitory receptors (predominately CTLA-4 and PD-1). Overall, HCMV- and HIV-specific CD4 T cells exhibit similar inhibitory receptor expression profiles, which differ considerably from Mtb-specific CD4 T cells, regardless of HIV infection status (Figure 5).

In addition to our analysis of inhibitory receptor co-expression profiles, we also considered the differential expression of total BTLA, CTLA-4, and PD-1 on Mtb-, HCMV- and HIV-specific T cells (Figure S6). With the exception of HIV-infected individuals with active TB disease, Mtb-specific CD4 T cells from all groups expressed significantly higher levels of BTLA, compared to HCMV-specific CD4 T cells. By contrast, HCMV- and HIV-specific CD4 T cells were consistently characterized by significantly higher expression of PD-1, compared with Mtb-specific CD4 T cells, regardless of Mtb or HIV infection status (Figure S6). Taken together, these data indicate that while virus-specific CD4 T cells co-express high levels of CTLA-4 and PD-1, Mtb-specific CD4 T cells within the same individuals do not upregulate expression of multiple inhibitory receptors simultaneously.

## Discussion

A state of T cell dysfunction, including dampened effector functions, upregulated inhibitory receptor expression, and decreased proliferation has been described during chronic infections (33), yet the relationship between HIV infection and the phenotype and function of Mtb-specific CD4 T cell responses has not been fully elucidated. This study was conducted to evaluate the effect of HIV on the concurrent expression of the Ig superfamily T cell inhibitory receptors BTLA, CTLA-4, and PD-1 on total and Ag-specific CD4 T cells from individuals with LTBI and active TB disease. We demonstrated that active TB disease in both HIV-uninfected and HIV-infected individuals is associated with reduced proportions of total CD4 T cells co-expressing three inhibitory receptors, compared with LTBI. Surprisingly, we found no evidence of increased expression of BTLA, CTLA-4, or PD-1, either alone or in combination, on Mtb-specific CD4 T cells in HIV-infected individuals, compared with HIV-uninfected individuals. Moreover, expression of BTLA was significantly lower on Mtb-specific CD4 T cells from HIV-infected individuals with active TB, compared with the other three participant groups. By directly comparing inhibitory receptor phenotypes of Mtb-, HCMV-, and HIV-specific CD4 T cells circulating concurrently within the same individual, we provide compelling evidence that, by contrast with virus-specific CD4 T cells, Mtb-specific CD4 do not co-express inhibitory receptors at high levels, regardless of HIV infection status.

Mtb-specific CD4 T cell production of the Th1 cytokines IFN-γ and TNF-α is important for activating macrophages and promoting formation of granulomas in the lung for containment of Mtb (34). By stimulating whole blood with PPD, we have previously found that HIV-infected individuals with LTBI had lower frequencies of Th1 cytokine-producing CD4 T cells than HIV-uninfected individuals with LTBI (5), consistent with previous reports (11, 12). While PPD contains >150 Ags (35, 36), in this study we used peptide pools spanning two immunodominant Ags to identify Mtb-specific CD4 T cells. By focusing on CFP-10/ESAT-6-specific CD4 T cells, we found similar frequencies of IFN-γ and/or TNF-α-producing CD4 T cells in HIV-infected and HIV-uninfected individuals with either LTBI or active TB disease, consistent with previous studies (37–39). In addition to Ag specificity, another important consideration in determining whether or not Mtb-specific CD4 T cells are depleted in HIV-infected individuals is the number and type of cytokines measured. While production of Th1 cytokines, including IFN-γ, TNF- α, and IL-2 are commonly evaluated in studies of Ag-specific CD4 T cells, we did not include IL-2 in this study to identify Mtb-specific CD4 T cells for phenotypic analysis. IL-2 is important for T cell survival and is one of the first cytokines that is lost by dysfunctional Ag-specific T cells in chronic infections (33). A recent study of individuals with LTBI and active TB in Tanzania revealed that HIV infection is associated with reduced proportions of Th2 and IL-2-producing Mtb-specific CD4 T cells (10), thus highlighting the importance of evaluating multiple different cytokines to more comprehensively evaluate the effect of HIV co-infection on Mtb-specific T cell immune function. It is possible that future studies measuring CD4 T cells specific for Ags other than CFP-10 and ESAT-6, and that include measurement of additional cytokines, such as additional Th1 cytokines as well as Th2 and Th17 cytokines, may reveal additional information on the frequency and phenotype of Mtb-specific CD4 T cells that differentiate HIV-infected and HIV-uninfected individuals.

Although we did not find evidence of preferential depletion of CFP-10/ESAT-6-specific CD4 T cells in HIV-infected individuals, evaluation of phenotypic markers, such as the inhibitory receptors BTLA, CTLA-4, and PD-1, provide additional insight into the functional nature of Ag-specific T cells. Multiple inhibitory receptors have been implicated as key contributors to the loss of immune control in cancer and chronic viral infections (19, 40), yet the role of inhibitory receptors immune control of Mtb is less clear. Given that HIV-infected individuals are at substantially higher risk of developing active TB, compared with HIV-uninfected individuals, we initially hypothesized that inhibitory receptors may be upregulated on Mtb-specific CD4 T cells in HIV-infected individuals, thus identifying a possible mechanism contributing to Mtb-specific CD4 T cell dysfunction in HIV infection. We were specifically interested in determining whether Mtb-specific CD4 T cells co-expressed multiple inhibitory receptors in HIV-infected individuals, as concurrent expression of multiple inhibitory receptors on the same Ag-specific T cell has been previously associated with greater T cell dysfunction (17, 22). While previous studies have reported co-expression of up to three inhibitory receptors on HIV-specific CD4 T cells (21–23), co-expression of multiple inhibitory receptors has not been thoroughly investigated on Mtb-specific CD4 T cells. Contrary to our initial hypothesis, we found that co-expression of all three inhibitory receptors was significantly lower on Mtb-specific CD4 T cells in HIV-infected individuals with active TB disease, compared with HIV-uninfected individuals with active TB. Interestingly, HIV infection did not have a significant impact on inhibitory receptor expression profiles of Mtb-specific CD4 T cells among individuals with LTBI. These data suggest that the combination of active TB disease and HIV infection together modify the phenotypic profiles of Mtb-specific CD4 T cells. However, it is important to note that we defined Mtb-specific CD4 T cells as those cells producing IFN-γ and/or TNF-α following short-term peptide stimulation, and it is possible that other approaches to defining Mtb-specific T cells (i.e., different Ag specificity, different effector functions, or direct MHC tetramer staining without peptide stimulation) may reveal differences in inhibitory receptor expression by Mtb-specific T cells than we observed in this study. Furthermore, increasing evidence indicates that age, sex, and genetics contribute significantly to heterogeneity in immune response profiles in humans (41, 42), thus additional differences in Mtb-specific T cell responses may emerge in studies utilizing cohorts from diverse geographical regions.

Through Boolean analysis of BTLA, CTLA-4, and PD-1 expression on Mtb-specific CD4 T cells, we identified three distinct BTLA-expressing subsets that are significantly lower in HIV-infected active TB patients, compared with HIV-uninfected TB patients: BTLA^+^CTLA-4^+^PD-1^+^, BTLA^+^CTLA-4^+^PD-1^−^, and BTLA^+^CTLA-4^−^PD-1^−^ cells. We found that BTLA expression is downregulated on bulk CD4 T cells in HIV-uninfected and HIV-infected individuals with active TB, a finding that is consistent with a previous report that BTLA expression is progressively downregulated on CD4 and CD8 T cells in chronic HIV infection (32). However, when evaluating Mtb-specific CD4 T cells, we found that BTLA was markedly downregulated only in HIV-infected patients with active TB, compared with all three other study groups. Thus, these data identify a novel phenotype of Mtb-specific CD4 T cells that is particular to the dual combination of active TB and HIV infection. The identification of potential biomarkers that can distinguish individuals with latent and active TB is particularly important in the setting of HIV infection, where HIV-infected individuals are less likely to have cavitary TB disease and less likely to be smear-positive for Mtb, compared with HIV-uninfected individuals (43), thus making microbiologically confirmed diagnosis of active TB disease particularly challenging in people living with HIV. Larger prospective, longitudinal studies of HIV-infected individuals are warranted to determine whether downregulation of BTLA expression on Mtb-specific CD4 T cells is a prognostic indicator of active TB disease, or whether BTLA expression is downregulated only after exposure to high levels of Mtb Ag in the context of HIV infection.

The role of BTLA expression in T cell-mediated immune control of Mtb infection has not been investigated and requires further study. BTLA was defined as an inhibitory receptor containing two immunoreceptor tyrosine-based inhibition motifs (ITIMs) in its cytoplasmic tail that is functionally and structurally similar to CTLA-4 and PD-1 (44). BTLA is expressed on multiple immune cell types and interacts with the costimulatory molecule herpes virus entry mediator (HVEM) (45, 46), which is also widely expressed on immune cells. Increasing evidence indicates bidirectional signaling occurs through the interaction between BTLA and HVEM, with HVEM ligation of BTLA leading to inhibitory signals through phosphorylation of ITIMs and recruitment of the tyrosine phosphatases SHP1 and SHP2 (44, 47), and BTLA ligation of HVEM leading to proinflammatory signals through activation of NF-κB (48), thus providing a unique opportunity for BLTA and HVEM engagement to balance resulting immune responses. Studies in mice indicate that BTLA expression is necessary to prevent prolonged inflammation in the lung (49) and that BTLA-deficient CD4 T cells have altered expression of genes involved in effector function and memory differentiation, including decreased expression of CD127, granzyme B, MIP-1α, and MIP-1β (50). Moreover, generation of protective memory Ag-specific CD8 T cell responses was significantly impaired in vaccinia virus-infected mice deficient in either BTLA or HVEM (51). Taken together, these data suggest co-signaling of BTLA and HVEM may regulate inflammatory responses in tissues and may also be necessary for generation of long-lived Ag-specific memory T cell responses. Further studies are thus warranted to determine whether lack of BTLA expression on Mtb-specific CD4 T cells in HIV-infected individuals with active TB disease is associated with a sustained state of inflammation in these individuals, and/or with impaired ability to generate robust Mtb-specific memory CD4 T cell responses in HIV-infected individuals. Future studies are also necessary to determine whether modulation of BTLA/HVEM co-signaling can fine tune the immune response to Mtb to promote durable control of infection and prevent progression to TB disease.

While we found compelling evidence that BTLA expression is downregulated on Mtb-specific CD4 T cells in HIV-infected patients with active TB, we found no evidence that CTLA-4 and PD-1 are upregulated in Mtb-specific CD4 T cells in HIV-infected individuals, unlike previous studies that have reported upregulation of CTLA-4 and PD-1 on HIV-specific CD4 T cells in HIV-infected individuals (52). To further substantiate our Mtb-specific CD4 T cell inhibitory receptor profiles in the context of the literature on inhibitory receptor profiles in HIV infection, we directly compared expression profiles of BTLA, CTLA-4, and PD-1 on Mtb-, HIV-, and HCMV-specific CD4 T cells within the same individual. Consistent with previous reports (21–23), HIV- and HCMV-specific CD4 T cells co-express CTLA-4 and PD-1, in both groups of LTBI and active TB, at significantly higher levels than Mtb-specific CD4 T cells. The direct comparison of inhibitory receptor profiles across multiple Ag-specific CD4 T cells circulating in peripheral blood provides compelling and novel evidence that the majority of Mtb-specific CD4 T cells do not upregulate co-expression of multiple inhibitory receptors, regardless of HIV infection status, and that Ag-specific CD4 T cells circulating in HIV-infected individuals display markedly different phenotypic profiles, depending on the Ag specificity.

A limitation to our study was the use of overlapping peptide pools to stimulate IFN-γ and/or TNF-α production to identify Ag-specific CD4 T cells, which precludes our ability to detect Mtb-specific cells that do not produce these cytokines and thus may display different inhibitory receptor profiles than Ag-specific CD4 T cells that maintain Th1 cytokine production capacity. Nonetheless, our findings are consistent with a previous study using MHC class II tetramers bearing CFP-10 and ESAT-6 peptides, which demonstrate low levels of PD-1 expression on tetramer^+^ Mtb-specific CD4 T cells in HIV-infected and HIV-uninfected individuals with LTBI and with active TB (39). An additional limitation was evaluation of inhibitory receptor expression on Mtb-specific CD4 T cells circulating in peripheral blood, and not at the site of Mtb infection in the lung. While analysis of lung-resident T cells is technically challenging in humans, a recent study has evaluated expression of inhibitory receptors on T cells isolated from granulomas of Mtb-infected macaques. Consistent with our findings in peripheral blood of humans, expression of the inhibitory receptors CTLA-4, PD-1, and LAG-3 was very low on T cells isolated from granulomas of Mtb-infected macaques and did not correlate with Mtb bacterial load (53). Furthermore, while we evaluated expression of BTLA, CTLA-4, and PD-1 in this study, there are additional inhibitory receptors, such as T cell immunoglobulin and mucin domain-containing molecule 3 (TIM-3) and Lag-3, that could be differentially expressed on Mtb-specific CD4 T cells in HIV-infected and HIV-uninfected individuals. Interestingly, expression of TIM-3 on CD4 and CD8 T cells in the lungs of Mtb-infected mice has been associated with T cell exhaustion (54), whereas TIM-3 expression on CD4 and CD8 T cells in PBMCs from patients with active TB disease exhibited greater Th1 cytokine production capacity and cytotoxic molecule production, compared with T cells lacking TIM-3 expression (55). These studies thus highlight the variability in the functional significance of inhibitory receptor expression in Mtb infection, which can differ depending on the particular inhibitory receptor, cell populations evaluated, and the model system used. Future studies employing RNA sequencing of Mtb-specific CD4 T will be necessary to more comprehensively define CD4 T cell signatures of latent and active TB, and further define how co-infection with HIV impairs protective T cell immunity to Mtb infection. An additional important consideration in evaluating the effect of HIV co-infection on Mtb-specific T cell immunity is HIV disease state. While the HIV-infected participants in our cohorts had relatively preserved CD4 T cell counts, it is possible that more substantial differences in inhibitory receptor expression on Mtb-specific CD4 T cells would be apparent in HIV-infected individuals with CD4 T cell counts <200 cells/μl and more advanced HIV disease.

In summary, by evaluating concurrent expression of the Ig superfamily inhibitory receptors BTLA, CTLA-4, and PD-1, we determined that these inhibitory receptors are not upregulated on Mtb-specific CD4 T cells in peripheral blood of HIV-infected individuals, compared with HIV-uninfected individuals, in the context of either LTBI or pulmonary TB disease. These data suggest that the increased risk of developing active TB disease in HIV-infected individuals is not due solely to upregulation of inhibitory receptors and subsequent immune exhaustion of Mtb-specific CD4 T cells, a mechanism of Ag-specific T cell dysfunction that has been well-described in other persistent infections (19, 40). Moreover, we provide evidence that BTLA is markedly downregulated on Mtb-specific CD4 T cells in HIV-infected individuals with active TB, thus highlighting a previously unrecognized role of BTLA expression levels on Mtb-specific CD4 T cells as a potential biomarker of active TB disease, particularly in people living with HIV. Together these data provide new insights into the phenotype of Mtb-specific CD4 T cells in the setting of co-infection with Mtb and HIV and provide rationale for future studies to evaluate the utility of targeting BTLA and HVEM signaling pathways to enhance protective immunity to Mtb.

## Data Availability

All datasets analyzed for this study are included in the manuscript/Supplementary Files.

## Author Contributions

CD and MB contributed conception and design of the study and data interpretation, statistical analyses, and drafted the manuscript. MB, DA, JK, JO, and JT performed experimental work. CD, MB, SO, FO, MdK, AC, NG, and WH contributed to execution and oversight of experimental work, participant recruitment and enrollment, and study database management. All authors approved the final manuscript.

### Conflict of Interest Statement

The authors declare that the research was conducted in the absence of any commercial or financial relationships that could be construed as a potential conflict of interest.
