# Supplementary material for: HIV Infection Is Associated With Downregulation of BTLA Expression on Mycobacterium tuberculosis-Specific CD4 T Cells in Active Tuberculosis Disease
Source: Front Immunol. 2019 Aug 21;10:1983. doi: 10.3389/fimmu.2019.01983 (PMC6712065; doi:10.3389/fimmu.2019.01983)
Supplement: Supplementary file 1 [file Data_Sheet_1.PDF]

Supplementary Material

HIV infection is associated with downregulation of BTLA expression on *Mycobacterium tuberculosis*-specific CD4 T cells in active tuberculosis disease

Morgan S. Barham, Deborah A. Abrahams, Jeremiah Khayumbi, Joshua Ongalo, Joan Tonui, Angela Campbell, Marwou de Kock, Samuel Gurrion Ouma, Felix Hayara Odhiambo, Willem A. Hanekom, Neel R. Gandhi, Cheryl L. Day*[*]

[*]**Correspondence**: Cheryl L. Day: cday@emory.edu

1. Supplementary Figures

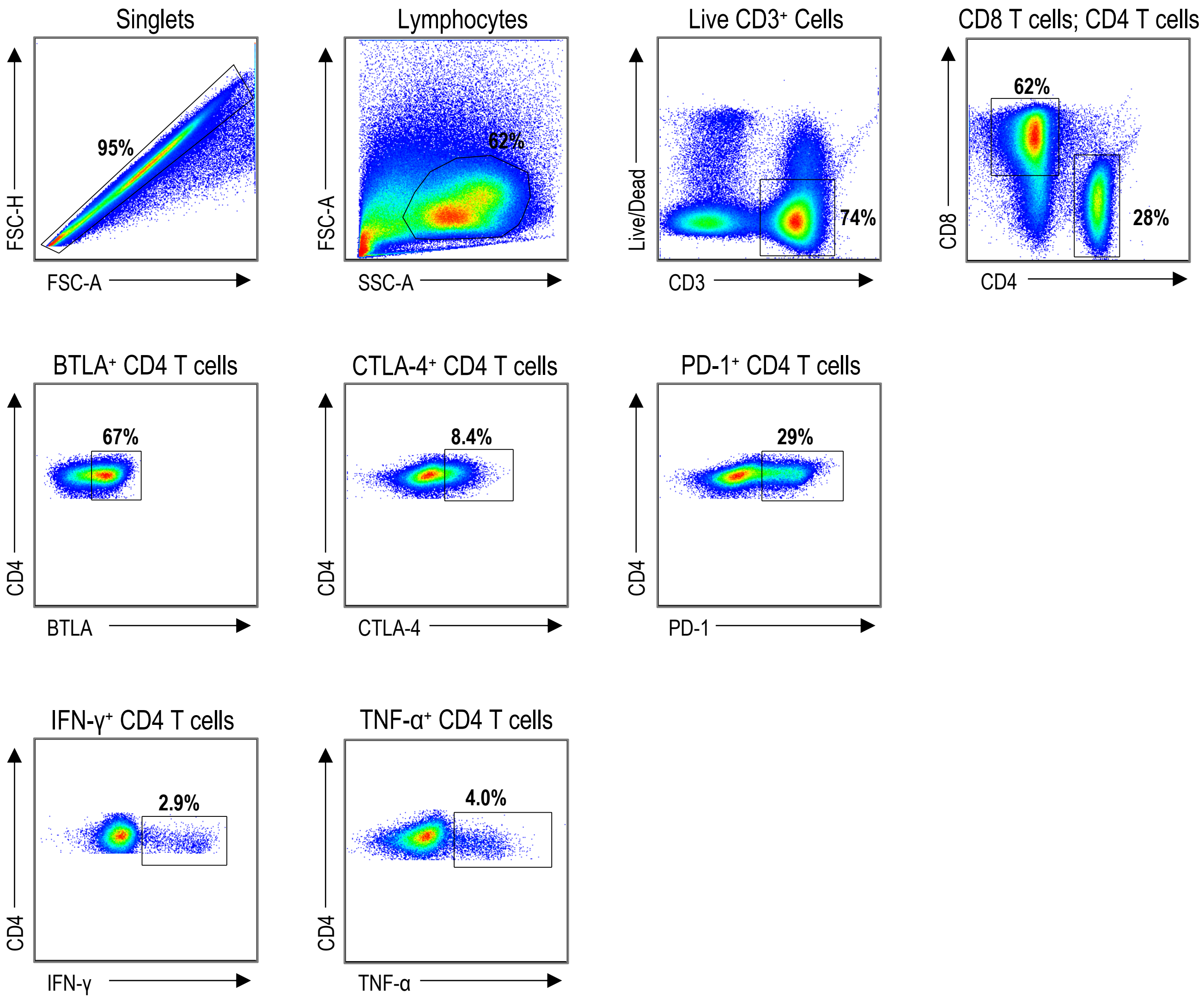

Figure S1. Gating strategy for flow cytometry analysis. In this sample gating, cells were first gated for singlets (FSC-H vs. FSC- A) and lymphocytes (SSC-A vs. FSC-A). The lymphocyte gate is further analyzed for their uptake of the Zombie IR Live/Dead stain to determine live versus dead cells and their expression of CD3 (Zombie NIR^{lo}, CD3^{+}). CD4 and CD8 surface expression is then determined from this gated population, followed by inhibitory receptor (BTLA, CTLA-4, and PD-1) expression and intracellular cytokine production (IFN-γ and TNF-α). Cytokine production is shown by CD4 T cells following stimulation with SEB.

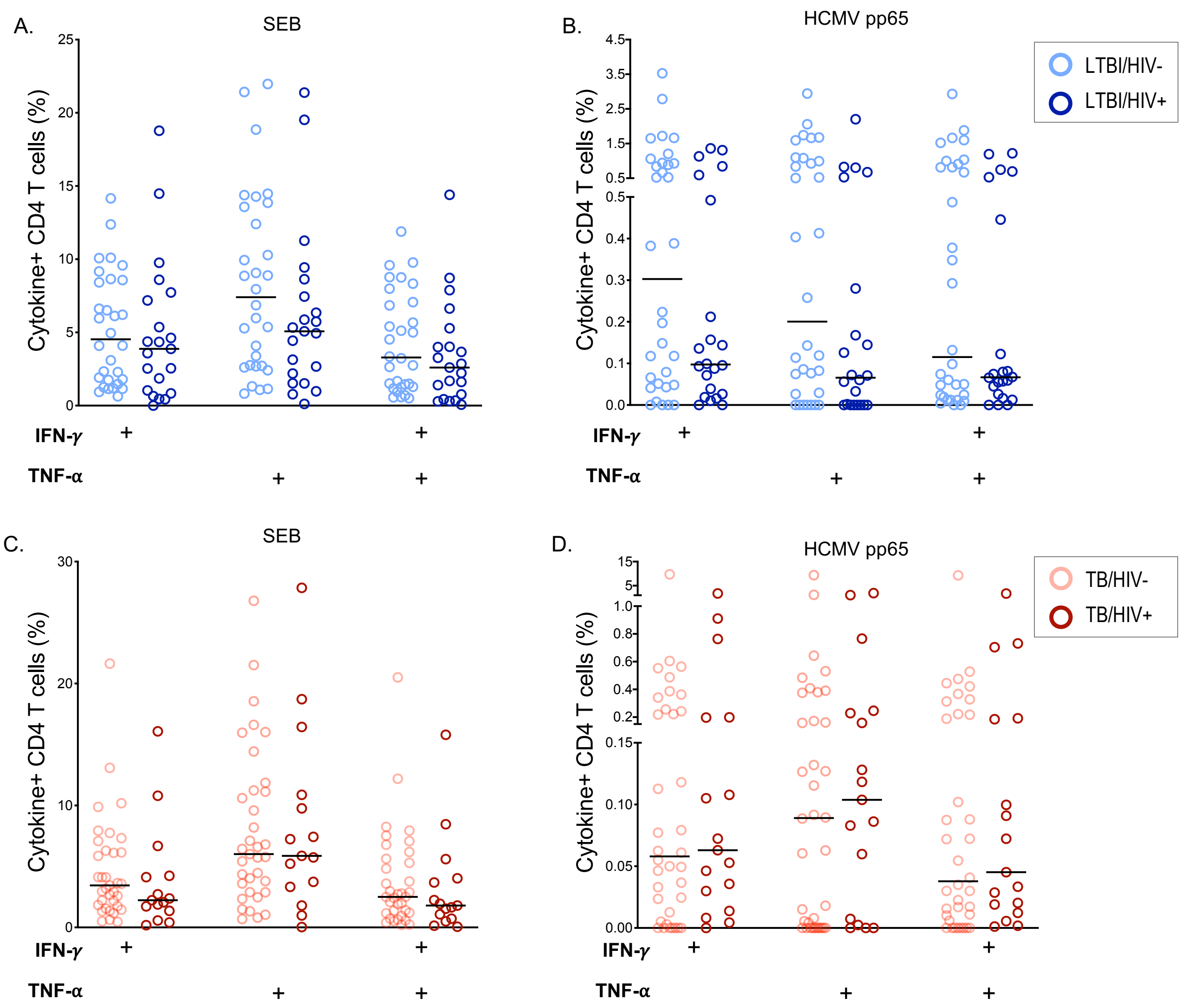

Figure S2. Similar frequencies of SEB and HCMV-specific T cells producing IFN-γ and TNF-α in HIV-infected and HIV-uninfected individuals with latent and active TB. PBMCs from HIV-uninfected and HIV-infected individuals with LTBI and HIV-uninfected and HIV-infected individuals with active TB were incubated for 6 hours with SEB (positive control) or HCMV pp65 peptide pools. Intracellular expression of IFN-γ and TNF-α was measured by flow cytometry. Frequencies of the indicated cytokine+ subsets of (A) SEB-stimulated and (B) HCMV-specific CD4 T cells from HIV-uninfected and HIV-infected individuals with LTBI. Frequencies of the indicated cytokine+ subsets of (C) SEB-stimulated and (D) HCMV-specific CD4 T cells from HIV-uninfected and HIV-infected individuals with active TB disease. Horizontal lines represent the median. Data are shown after subtraction of background cytokine production in the unstimulated negative control condition. Differences in the frequencies of each cytokine+ T cell population between HIV-uninfected and HIV-infected individuals were assessed using a Mann Whitney *U* test; no significant differences were found between HIV-uninfected and HIV-infected groups.

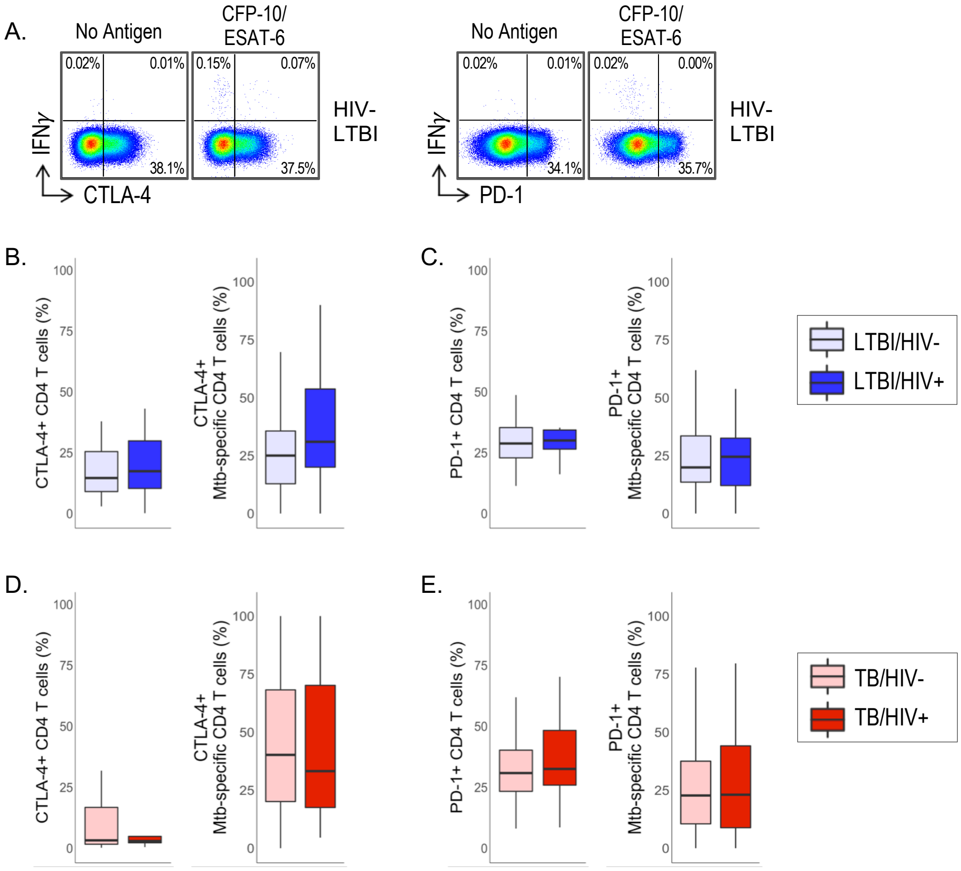

Figure S3. CTLA-4 and PD-1 expression on Mtb-specific CD4 T cells in latent and active TB. PBMCs from HIV-uninfected and HIV-infected individuals with LTBI and TB were either left unstimulated or stimulated with CFP-10 and ESAT-6 peptide pools as described in Figure 1. (A) Representative flow cytometry data from an HIV-uninfected individual with LTBI. Plots are shown gated on live $CD3^{+}CD4^{+}$ T cells. (B, C) Composite data of the percentage of CTLA-4 (B) and PD-1 (C) expression on total unstimulated CD4 T cells and CFP-10/ESAT-6-specific CD4 T cells from individuals with LTBI. (D, E) Composite data of the percentage of CTLA-4 (D) and PD-1 (E) expression on total unstimulated CD4 T cells and CFP-10/ESAT-6-specific CD4 T cells from individuals with active TB. Boxes represent the median and interquartile ranges; whiskers represent the 5th and 95th percentiles. Differences in the percentages between HIV-uninfected and HIV-infected individuals were assessed using a Mann-Whitney *U* test.

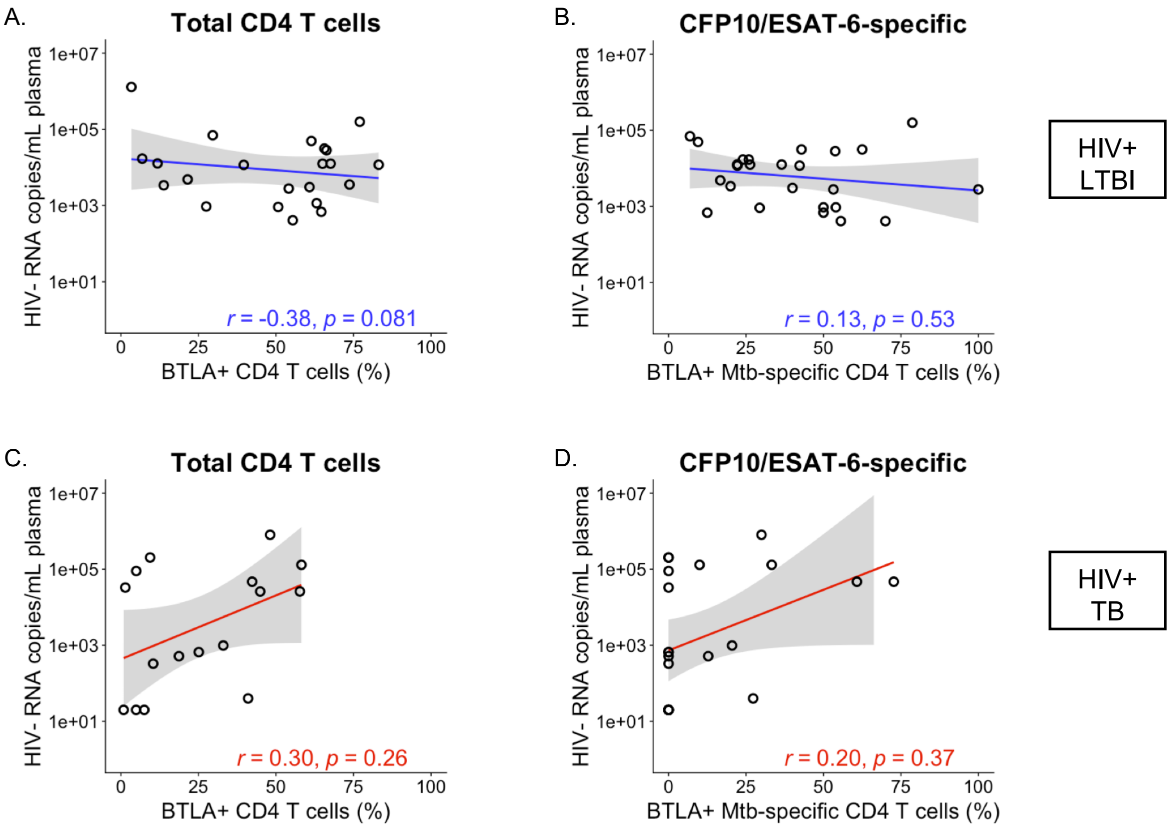

Figure S4. BTLA expression does not correlate with HIV viral load in HIV-infected individuals with LTBI and active TB. PBMCs from HIV-infected individuals with LTBI and TB were either left unstimulated or stimulated with CFP-10 and ESAT-6 peptide pools as described in Figure 1. (A, B) Correlation between HIV viral load and BTLA expression on bulk CD4 T cells (A) and Mtb-specific CD4 T cells (B) in HIV-infected individuals with LTBI. (C, D) Correlation between HIV viral load and BTLA expression on bulk CD4 T cells (A) and Mtb-specific CD4 T cells (B) in HIV-infected individuals with active TB. Statistical significance was evaluated using the non-parametric Spearman rank test.

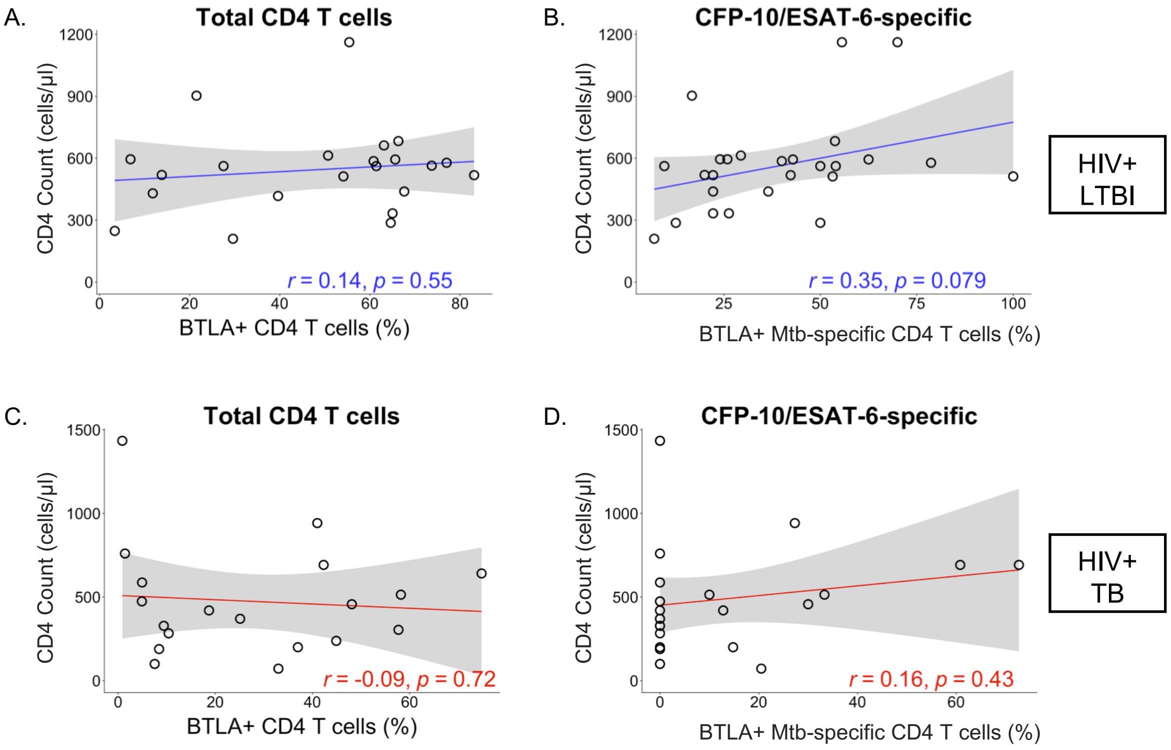

Figure S5. BTLA expression does not correlate with absolute CD4 T cell count in HIV-infected individuals with LTBI and active TB. PBMCs from HIV-infected individuals with LTBI and TB were either left unstimulated or stimulated with CFP-10 and ESAT-6 peptide pools as described in Figure 1. (A, B) Correlation between absolute CD4 T cell count and BTLA expression on bulk CD4 T cells (A) and Mtb-specific CD4 T cells (B) in HIV-infected individuals with LTBI. (C, D) Correlation between absolute CD4 count and BTLA expression on bulk CD4 T cells (C) and Mtb-specific CD4 T cells (D) in HIV-infected individuals with active TB. Statistical significance was evaluated using the non-parametric Spearman rank test.

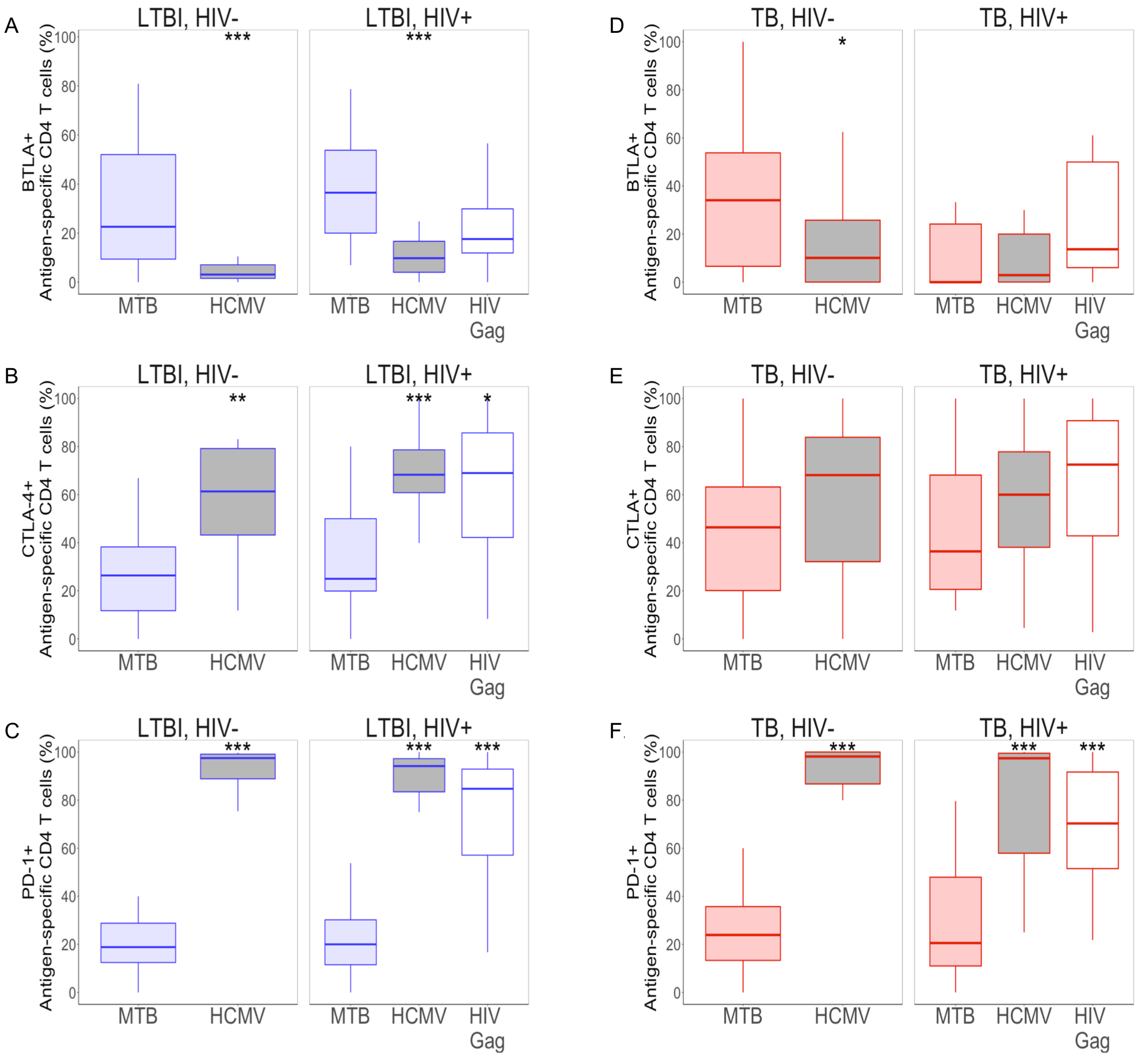

Figure S6. Mtb-specific CD4 T cells express higher levels of BTLA and lower levels of CTLA-4 and PD-1, compared with HCMV- and HIV-specific CD4 T cells. PBMCs were stimulated with peptide pools as described in Figure 1. Expression levels of BTLA, CTLA-4, and PD-1 on Ag-specific CD4 T cells in HIV-uninfected and HIV-infected individuals with LTBI are shown in panels A, B, and C, respectively, and in HIV-uninfected and HIV-infected individuals with active TB in panels D, E, and F, respectively. Boxes represent the median and interquartile ranges; whiskers represent the 5th and 95th percentiles. Differences in the inhibitory receptor expression of HCMV- or HIV-specific CD4 T cells was compared with Mtb-specific CD4 T cells using a Mann-Whitney *U* test. * $p < 0.05$; ** $p < 0.01$; *** $p < 0.001$.
